# Comparative virulence of diverse *Coxiella burnetii* strains

**DOI:** 10.1080/21505594.2019.1575715

**Published:** 2019-02-20

**Authors:** Carrie M Long, Paul A Beare, Diane C Cockrell, Charles L Larson, Robert A Heinzen

**Affiliations:** Coxiella Pathogenesis Section, Laboratory of Bacteriology, Rocky Mountain Laboratories, National Institute of Allergy and Infectious Diseases, National Institutes of Health, Hamilton, MT, USA

**Keywords:** *Coxiella burnetii*, Q fever, virulence, guinea pig model, gram-negative bacteria, intracellular bacteria

## Abstract

*Coxiella burnetii* is an intracellular, gram-negative bacterium that causes the zoonosis Q fever. This disease typically presents as an acute flu-like illness with persistent, focalized infections occurring less frequently. Clinical outcomes of Q fever have been associated with distinct genomic groups of *C. burnetii*, suggesting that gene content is responsible for virulence potential. To investigate this hypothesis, the virulence of thirteen *C. burnetii* strains (representing genomic groups I-VI) was evaluated in a guinea pig infection model by intraperitoneal injection. Seven strains caused a sustained fever (at least two days ≥39.5°C) in at least half of the animals within each experimental group. At fourteen days post infection, animals were euthanized and additional endpoints were evaluated, including splenomegaly and serology. The magnitude of these endpoints roughly correlated with the onset, duration, and severity of fever. The most severe disease was caused by group I strains. Intermediate and no virulence were evidenced following infection with group II-V and group VI strains, respectively. Flow cytometric analysis of the mesenteric lymph nodes revealed decreased CD4^+^ T cell frequency following infection with highly virulent group I strains. These findings buttress the hypothesis that the pathogenic potential of *C. burnetii* strains correlates with genomic grouping. These data, combined with comparative genomics and genetic manipulation, will improve our understanding of *C. burnetii* virulence determinants.

## Introduction

*Coxiella burnetii* is a gram-negative, intracellular bacterium with worldwide dissemination [1]. This bacterium is clinically significant due to its identity as the causative agent of the zoonosis Q fever. Because of high infectivity, environmental stability, aerosol transmission, and the debilitating nature of Q fever, *C. burnetii* is considered a potential biological weapon, resulting in its classification as a select agent [2]. Dairy cows, goats, and sheep are the primary reservoirs responsible for human infection which typically occurs following inhalation of infectious aerosols derived from these animals and their products. Q fever generally presents as an acute illness marked by flu-like symptoms and high fever, although many individuals remain asymptomatic throughout infection. Full recovery is common following acute illness, particularly after antibiotic treatment. However, some patients may develop persistent focalized infections (formerly referred to as “chronic Q fever”) such as endocarditis, hepatitis, lymphadentitis, myocarditis, osteomyelitis, and/or vascular infection [3,4].

Many *C. burnetii* strains have been isolated since the initial recognition of the bacterium in the late 1930s [5–7]. Correlations have been made between strains and disease type (e.g. acute vs persistent focalized infections). Indeed, the concept of *C. burnetii* pathotypes arose from observations that isolates from acute or persistent infections group according to genome content as well as lipopolyscharide (LPS) chemotype [7,8]. Samuel et al. [9] investigated the relationship between *C. burnetii* plasmid type and the natural origin of each strain, reporting correlations between plasmid types and strains which cause acute human disease and those that cause chronic endocarditis. A seminal study by Hendrix et al. [7] compared several *C. burnetii* strains via restriction endonuclease digestion pattern analysis of genomic DNA, resulting in the designation of six distinct genomic groups which showed a pattern of association with acute or persistent focalized human disease. Genomic group I-III strains harbour the plasmid QpH1 and have been isolated from the blood of human acute Q fever patients, chiggers, cow’s milk, goat abortions, and ticks. Strains within group IV contain QpRS and are derived from the heart valves of Q fever endocarditis patients and livestock abortion products. Group V strains do not have a plasmid; rather, plasmid-like sequences are contained within the chromosome. These strains were collected from human Q fever endocarditis or hepatitis patients. Lastly, group VI strains, originating from rodents in the Utah desert, contain QpDG, and display severely attenuated virulence [10,11]. Both contradictory and confirmatory evidence of plasmid-disease associations were provided by Glazunova et al. [12] who performed multispacer sequence typing (MST) of 173 isolates. In this study, no correlation was found between QpH1 and disease type. However, correlations were found between QpDV and acute disease and QpRS and persistent focalized infections. QpDV is associated with new genomic groups VII and VIII as defined by Beare et al. [13]. Further studies using multiple-locus variable number of tandem repeats analysis and single nucleotide polymorphism typing of MST loci revealed similar correlations between genomic content and disease presentation [14–16].

All strains obtained from natural sources express full-length (phase I) LPS which is necessary for full virulence [17]. Indeed, phase I LPS is the only virulence factor of *C. burnetii* that has been defined in an immunocompetent animal model [18]. Phase I LPS is severely truncated following serial *in vitro* passage in cell culture, embryonated hen’s eggs, or synthetic medium, generating avirulent phase II organisms which coincides with a complete loss of virulence [18–22]. This process is referred to as phase variation. The truncated LPS of phase II bacteria lacks O-antigen and several additional core sugars [23]. Because some avirulent environmental strains express phase I LPS, additional factors likely contribute to virulence.

Undoubtedly, host and environmental conditions also influence disease outcome and clinical presentation of infection. Clinical studies support this idea, as both interleukin 10 and tumor necrosis factor-alpha production appear to be linked to the occurrence of Q fever endocarditis [24–26]. Both valvular disease and immunosuppression are known risk factors for Q fever endocarditis, emphasizing the importance of host factors in disease development [27]. Additionally, a case-control study conducted to evaluate potential risk factors involved in the recent Q fever outbreak in the Netherlands identified several major risk factors associated with the development of persistent focalized infections including, advancing age, aneurysms, renal insufficiency, valvular surgery, and vascular prosthesis [28]. Notably, gender has been demonstrated to impact *C. burnetii* infection as males account for approximately 75% of patients diagnosed with *C. burnetii* endocarditis and a higher ratio of males to females has been reported among hospitalized Q fever patients [29,30]. In mice, 17β-estradiol confers a protective effect when administered prior to *C. burnetii* infection [31] and circadian rhythm has been implicated in the disparate responses to infection displayed among female and male animals [30].

In an effort to better understand the relationship between genomic content and virulence potential, the present study assayed several *C. burnetii* strains for virulence in a guinea pig model of intraperitoneal infection. Strain virulence was evaluated by the examination of fever responses, body weight loss, splenomegaly, and other informative endpoints.

## Materials and methods

### Coxiella burnetii strains

Thirteen *C. burnetii* strains representing six genomic groups were utilized as inocula for guinea pig infections (Table 1). All strains were passaged in acidified citrate cysteine medium-D (ACCM-D) [32] for two week-long passages at 37°C in a 2.5% O_2_ and 5% CO_2_ environment prior to storage as inocula. Aliquots were stored in cell freezing media (Dulbecco's Modified Eagle Medium with 10% fetal bovine serum and 10% dimethyl sulfoxide) at −80°C until guinea pig infections. All manipulations of phase I *C. burnetii* stocks and infected animal tissue were performed in a Biosafety Level-3 (BSL-3) laboratory in accordance with BSL-3 standard operating procedures (SOPs) approved by the Rocky Mountain Laboratories Institutional Biosafety Committee.

**Table 1.** Description of *C. burnetii* strains used in this study.

Genomic Group [7]	Strain	Plasmid Type [7]	Origin	[Human] Disease Caused	Experiment Number^1^	Passage History^2^	LPS Type^3^
I	**Nine Mile I RSA493**	QpH1	Tick, Montana	Acute	1	307 GP/1 TC/1 E/2 TC/2 Med	I
I	**Nine Mile Crazy RSA514**	QpH1	Placental tissue from long-term NMI infection of guinea pig (343 days)	n/a	Ctrl	4 EP/343d GP/1 E/1 Med	Intermediate
I	**Nine Mile Crazy RSA514**	QpH1	Placental tissue from long-term NMI infection of guinea pig (343 days)	n/a	1	4 EP/343d GP/1 E/2 Med	Intermediate/II
I	**Nine Mile II RSA439**	QpH1	Tick, Montana then long-term passage *in vitro*	n/a	1	90 E/1 TC/1 E/1 Med	II
I	**Ohio RSA270**	QpH1	Cow’s Milk, Ohio	Persistent	1	4 E/2 Med	I
II	**Henzerling RSA343**	QpH1	Human Blood, Italy	Acute	2	6 GP/25 E/1 GP/4 E/2 Med	I
II	**Henzerling RSA331**	QpH1	Human Blood, Italy	Acute	2	6 GP/25 E/1 GP/36 EP/2 Med	I/II
III	**Idaho Goat Q195**	QpH1	Goat Placenta, Idaho	Abortion	1	2 E/2 Med	I
IV	**P Q238**	QpRS	Human Heart Valve, California	Endocarditis	2	3 E/2 Med	I
IV	**MSU Goat Q177 (Priscilla)**	QpRS	Goat Cotyledon, Montana	Abortion	2	1 GP/4 E/2 Med	I
V	**G Q212**	Integrated plasmid sequence	Human Heart Valve, Nova Scotia	Endocarditis	2	2 E/4 TC/2 Med	I
V	**S Q217**	Integrated plasmid sequence	Human Liver Biopsy, Montana	Hepatitis	2	2 E/2 Med	I
VI	**Dugway 7D 77-80**	QpDG	Rodents, Utah	n/a	1	4 E/2 Med	I
VI	**Dugway 7E 65-68**	QpDG	Rodents, Utah	n/a	1	3 E/2 Med	I

^1^Both studies had a saline-treated negative control group; ctrl, experimental Nine Mile Crazy LPS control for LPS analysis

^2^E, embryonated hen’s eggs; GP, guinea pig; Med, media; TC, tissue culture

**Figure 1.**
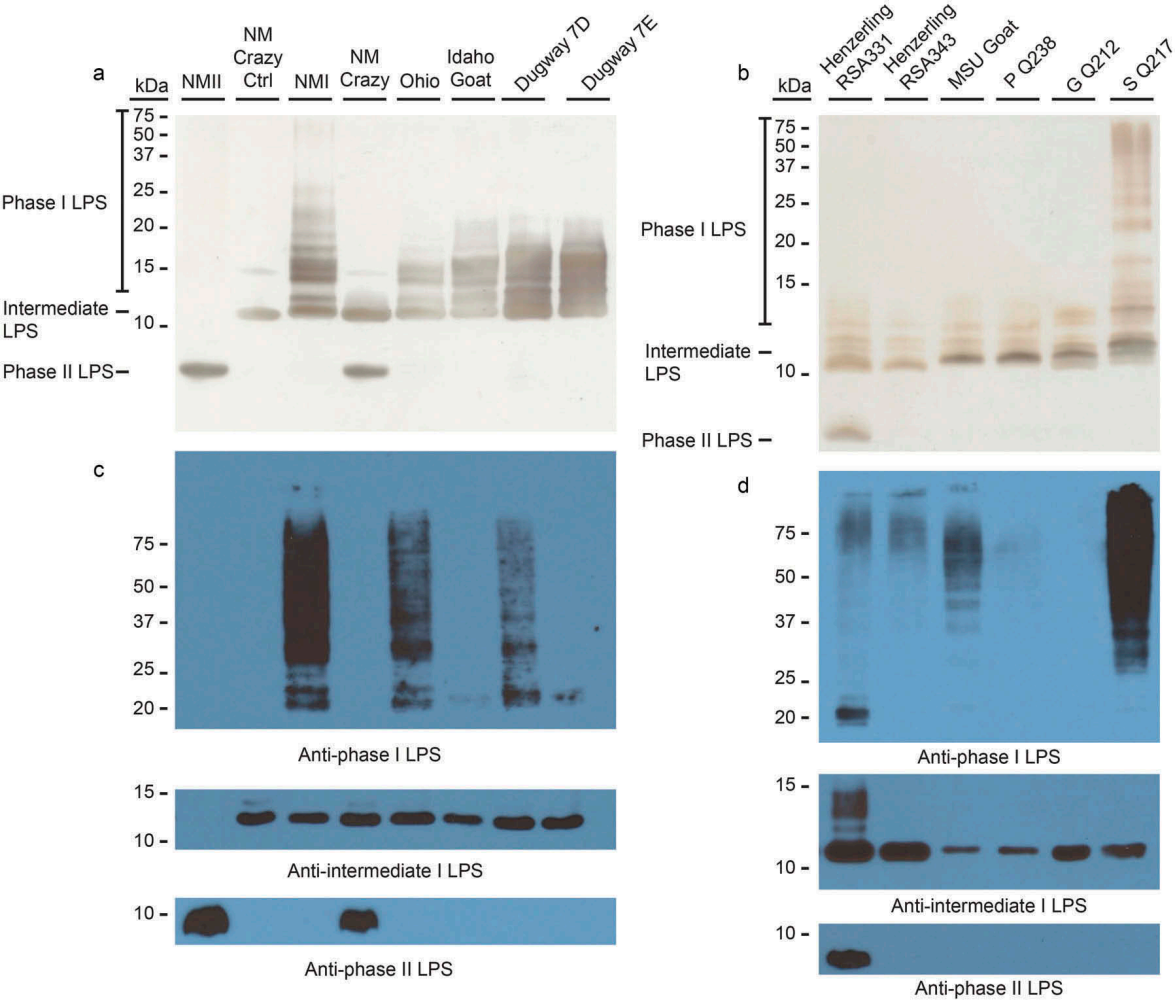
LPS phase profiling of *C. burnetii* strains used in this study. The LPS profile of each strain was determined by silver stain (a and b) and immunoblot (c and d). Silver stain banding corresponding to phase I, intermediate, and phase II LPS is indicated to the left of the gel (a and b). Immunoblots were conducted with anti-phase I, anti-intermediate, and anti-phase II LPS antibodies as described in the Materials and Methods [23]. NM Crazy ctrl represents the isogenic passage variant utilized as an LPS control, as previously described [23].

^3^Based on Figure 1

### Genome equivalent (GE) quantification of inocula

Aliquots of bacterial stocks in cell freezing media were boiled for 30 minutes (100°C) and diluted 1:20 in nuclease-free water. GE were quantified using TaqMan™ qPCR on a StepOnePlus Real-Time PCR system (Applied Biosystems) to determine the copy number of *C. burnetii groEL* [33,34].

### LPS extraction and visualization

*C. burnetii* LPS was extracted using a modified hot phenol method as previously described [13,23]. Extracted LPS was visualized by silver stain and immunoblot [23]. For silver staining, samples were separated by sodium dodecyl sulfate-polyacrylamide gel electrophoresis (SDS-PAGE) on 16% Tricine gels. In gel LPS was stained using the SilverQuest™ staining kit (ThermoFisher Scientific, cat. n. LC6070) according to the manufacturer’s instructions. For immunoblotting, LPS was separated by SDS-PAGE on 12 or 16% glycine gels and transferred to a polyvinylidene fluoride membrane. The following monoclonal primary antibodies were utilized for *C. burnetii* LPS recognition via immunoblotting: AB-COX-MAB1 (phase I LPS; BEI Resources, cat. n. DD-265; 1:10000 dilution), IE4 [35] (intermediate LPS; 1:10000 dilution; a generous gift of Guoguan Zhang, University of Missouri-Columbia), and A6 [23,36] (phase II LPS; 1:50 dilution). Goat anti-mouse IgG-horseradish peroxidase secondary antibody, along with Supersignal West Pico chemiluminescent substrate (ThermoFisher Scientific, cat n. 31430 and 34577), were utilized to detect reacting LPS. LPS bands were sized using the Precision Plus Dual Color protein ladder (Bio-Rad, cat. n. 1610374).

### Guinea pigs

Female 4-to-6 week old Hartley guinea pigs were purchased from Charles River (strain code 051) and acclimated for at least one week prior to experimental manipulation in order to minimize stress effects. In accordance with historical Q fever virulence studies, and in order to minimize confounding factors based on gender associated with study endpoints (e.g. body weight, hormonal effects, intraspecies aggression), female guinea pigs were utilized in these studies. Guinea pigs were housed in individually ventilated plastic cages (Allentown; two animals per cage) with hardwood chip bedding (Sani-chips; PJ Murphy). An Envigo lab animal diet (global high fiber guinea pig diet; Teklad, cat n. 2041) and chlorinated tap water (filtered by reverse osmosis) were administered *ad libitum*. Housing facilities were maintained at 20–22°C and 40–60% relative humidity (with a 50% set point), and a 12-h light–dark cycle was followed. All animals were housed an approved animal biosafety level 3 (ABSL-3) facility. All animal experiments and procedures were performed in the Association for Assessment and Accreditation of Laboratory Animal Care-accredited National Institutes of Health-National Institute of Allergy and Infectious Diseases animal facility. An Institutional Animal Care and Use Committee-approved protocol (ASP 2017–006-E) was used with ABSL-3 SOPs approved by the Rocky Mountain Laboratories Institutional Biosafety Committee.

### Experimental infection

On the day of infection, animals were sedated by isoflurane using an anesthetic vaporizer with activated charcoal adsorption filters (VetEquip Inc, cat. n. 901801 and 931401) and an IPTT-300 transponder (BioMedic Data Systems) was implanted subcutaneously above the shoulder of each animal in a longitudinal orientation using a large bore needle. Four guinea pigs per group were infected with 10^5^ GE of *C. burnetii* in United States Pharmacopeia-grade saline via intraperitoneal injection. Four negative control animals were mock infected with United States Pharmacopeia-grade saline for each experiment. Body weight, body temperature, and behavioral and clinical changes were recorded daily. Body temperatures were collected using a DAS-8007-P reader (BioMedic Data Systems) and a temperature of ≥39.5°C was defined as fever [37–39]. Body weight index (BWI) was calculated as defined by Russell-Lodrigue et al. [37].

### Euthanasia, tissue collection, and processing

Guinea pigs were euthanized at 14 days post infection via intraperitoneal ketamine injection followed by exsanguination (cardiac puncture) and induction of pneumothorax. Blood was collected by cardiac puncture using Vacutainer® blood collection tubes and needles (BD). Following euthanasia, two mesenteric lymph nodes and the spleen were excised and placed into tubes containing sterile phosphate-buffered saline (PBS; Gibco, pH: 7.4, cat. n. 10010023). Lymph nodes were manually dissociated using the frosted ends of two microscope slides. Lymph node cellularity was determined using a Scepter automated cell counter (Millipore, cat. n. PHCC20060) with size exclusion parameters (6–36 μm). Following counting, lymph node cells were immediately aliquoted for flow cytometry. Spleens were dissociated using disposable 15 mL tissue grinders (VWR International, cat. n. 47732-446). Lymph node and spleen suspensions were stored at −20°C for subsequent analysis. Blood was centrifuged in Vacutainer® collection tubes and serum was collected and stored at −80°C for subsequent analysis. Prior to serologic analysis, serum was manually filtered using 0.1 um Millipore Durapore® PVDF syringe filters (cat. n. EW-81053-10).

### Flow cytometry

Single cell suspensions from the mesenteric lymph nodes were aliquoted into 96-well U-bottom plates at a minimum density of 10^6^ cells per well. Cells were washed in staining buffer (PBS + 1% bovine serum albumin) and resuspended in staining buffer containing a cocktail of fluorochrome-conjugated antibodies specific for guinea pig cell surface antigens, including CD4 (clone: CT7, fluorophore: RPE, BioRad, cat. n. MCA749PE) and CD8 (clone: CT6, fluorophore: FITC, BioRad, cat. n. MCA752F). Following surface staining, cells were washed in staining buffer and fixed overnight at 4°C using Cytofix (BD, cat. n. 554655). Following fixation, cells were washed in staining buffer and analyzed on an LSR II flow cytometer using FacsDiva software (BD Biosciences). Data analysis was performed with FlowJo 10.0 software (TreeStar Inc., Ashland, Oregon). A minimum of 50,000 events were captured for each sample. Numerical population values were calculated by applying subset frequencies to the total cell count obtained following lymph node homogenization. Single-stained compensation controls and fluorescence minus one staining controls were included to help set gating boundaries.

### Culture of C. burnetii from organ homogenates

For recovery of *C. burnetii* from the spleens of infected animals, 100 μL to 1 mL of spleen homogenate was added to 3 mL ACCM-D in 6-well flat bottom plates. Following one week of incubation at 37°C in a 2.5% O_2_ and 5% CO_2_ environment, whole cultures were passaged into T-75 flasks containing 25 mL ACCM-D. A third passage into T-75 flasks was performed if second passage growth was insufficient for LPS extraction. Samples were evaluated for *C. burnetii* growth under a light microscope and LPS was profiled for cultures exhibiting sufficient growth.

### Immunofluorescence assay (IFA)

*C. burnetii*-specific IgG titers were determined using a modified version of the Q Fever IFA IgG kit (Focus Diagnostics, cat. n. IF0200G). Q fever substrate slides, containing individual phase I and II *C. burnetii* antigen, were exposed to 25 μL guinea pig serum diluted in IgG sample diluent at a variety of dilutions. Positive (kit-supplied human serum and study-derived guinea pig serum) and negative controls (study-derived guinea pig serum) were utilized to semi-quantitatively determine titers, as specified by the manufacturer’s instructions. Following slide incubation in a humid chamber for 30 minutes at 37°C, slides were washed with PBS, submerged in PBS for 10 minutes, dipped in water, and allowed to air dry. IgG conjugate (fluorescein-labeled goat anti-human IgG; provided in the IFA kit) was added (25 μL) to each well previously treated with human sera. Goat anti-guinea pig IgG heavy and light chain antibody conjugated to fluorescein isothiocyanate (Abcam, cat. n. ab6904) diluted 1:500 in PBS was added (25 μL) to each well previously treated with convalescent guinea pig sera. Following incubation of slides in a humid chamber for 30 minutes at 37°C, slides were washed with PBS, submerged in PBS for 10 minutes, dipped in water, and allowed to air dry. Mounting media was added to dried wells and 24 × 60 mm coverslips were used to cover wells. Slides were viewed at a magnification of 400X on a Nikon Eclipse Ti2 inverted epifluorescent microscope. The reciprocal of the highest dilution that exhibited dim fluorescence (equivalent to that of the positive control at its reference endpoint titer) was defined as the endpoint titer. Geometric mean titer values were calculated using the “GEOMEAN” function in Microsoft Excel.

### Statistical analysis

Statistical analyses were conducted using GraphPad Prism version 7.0. Data were analyzed by an unpaired t-test with Welch’s correction comparing groups as indicated in the figure legends. All differences were considered statistically significant at p < 0.05. Representative significance symbols varied by figure, as indicated in the legend.

## Results

### C. burnetii LPS displays passage-dependent forms

The *C. burnetii* strains utilized in this study originated from locales across the globe and were isolated from a variety of organisms and disease states (Table 1). Several Nine Mile strains were utilized as reference strains due to their historic experimental use and known virulence including Nine Mile I RSA493 (NMI), Nine Mile Crazy RSA514 (NM Crazy), and Nine Mile II RSA439 clone 4 (NMII) [18]. These strains are all contained within genomic group I and were derived from the original Nine Mile isolate which was recovered from a Rocky Mountain wood tick in Montana in 1935 [6]. NM Crazy is a derivative of NMI which exhibits an intermediate length LPS and attenuated virulence *in vivo* compared to its phase I counterpart (NMI) [18]. This strain was recovered from the placental tissue of a guinea pig after a 343 day-long persistent infection [17]. In our studies, two NM Crazy RSA514 passage variants were utilized: one as an LPS control (NM Crazy ctrl) and one for animal infections (NM Crazy) which has an additional passage in axenic medium. NMII is an avirulent strain derived following 90 egg passages of the original Nine Mile strain. NMII produces severely truncated LPS and is the only *C. burnetii* strain approved for use at BSL-2. Additional strains examined in this work were: Ohio RSA270 (Ohio), Henzerling RSA331, Henzerling RSA343, Idaho Goat Q195 (Idaho Goat), P Q238, MSU Goat Q177 (MSU Goat), G Q212, S Q217, Dugway 7D 77-80 (Dugway 7D) and 7E 65-68 (Dugway 7E). Table 1 contains a description of all strains utilized in this study. Due to the inclusion of numerous strains, the study was split into two experiments (Experiments 1 and 2; Table 1), each with appropriate experimental controls.

We first characterized the LPS produced by *C. burnetii* strains utilized for guinea pig infections by silver stain and immunoblot. Immunoblots employed antibodies specific for phase I, intermediate (e.g. NM Crazy ctrl, Figure 1(a,c)), and phase II LPS [23]. Figure 1(a,c) depict LPS from strains used in experiment 1. Figure 1(b,d) depict LPS from strains used in experiment 2. Silver staining revealed variations in phase I O-antigen banding (>12 kDa) among phase I LPS-expressing *C. burnetii* strains as previously described [8,23]. With the exception of NMII, these strains also produced intermediate LPS (~11 kDa), which consists of lipid A, inner and outer core sugars, and one repeating O-antigen unit lacking virenose [23]. Only intermediate LPS was produced by NM Crazy ctrl whereas NM Crazy produced both intermediate and phase II LPS (~3 kDa), the latter comprised of lipid A and core sugars [23]. NMII produced phase II LPS while Henzerling RSA331 produced all three LPS forms. Correlative phase I banding variation was also observed via immunoblotting although O-antigen of some phase I LPS-expressing strains showed minimal to no reactivity with the phase I LPS monoclonal antibody (i.e. Idaho Goat, Dugway 7E, P Q238, and G Q212). Differential reactivity of O-antigen to antibody has been observed before and correlates with different banding patterns associated with genomic grouping observed by silver stain [8]. These data agree with previous reports that the appearance of phase II LPS correlates with *in vitro* passage [23].

### Temporal fever responses of guinea pigs infected with C. burnetii strains reveal a range of virulence

**Figure 2.**
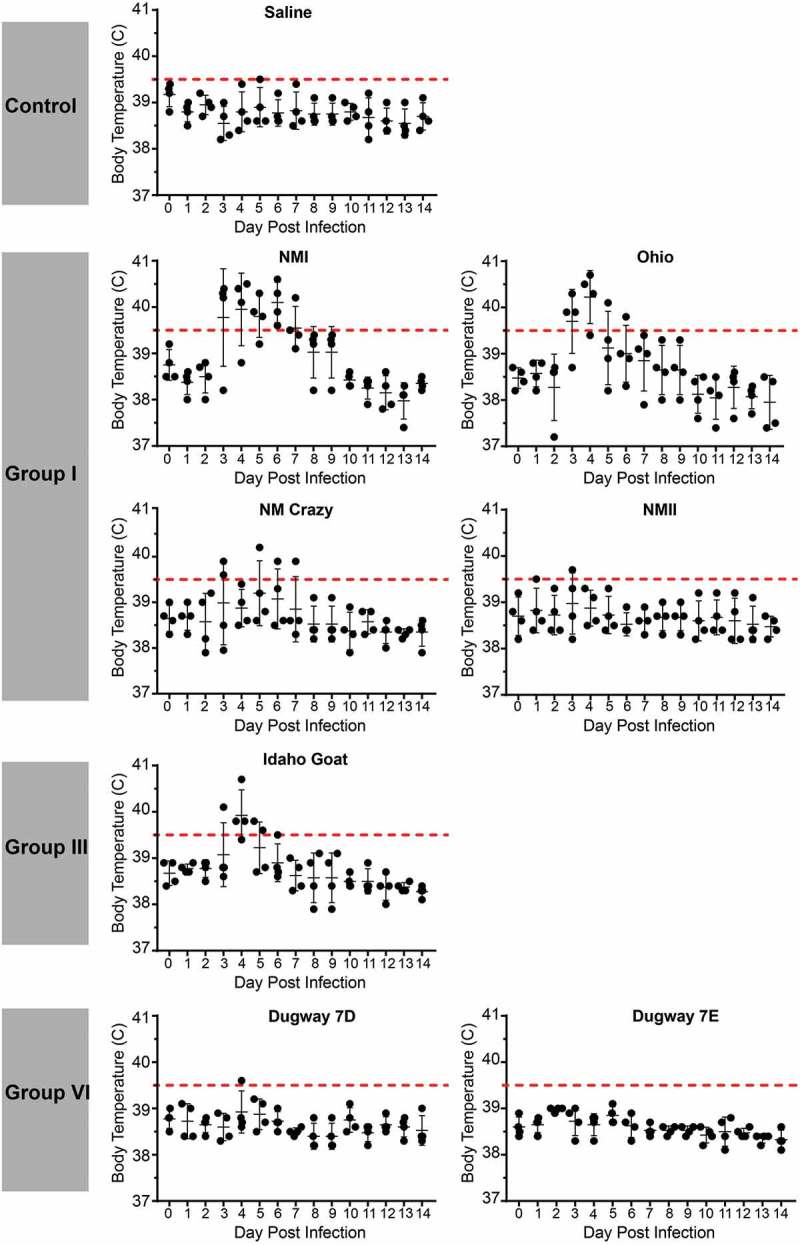
Fever responses of *C. burnetii*-infected guinea pigs in experiment 1. Body temperatures were recorded daily from the day of infection (day 0) until the day of euthanasia (day 14). Temperatures were plotted for each individual animal per time point. Fever was defined as ≥ 39.5°C and is indicated by the dotted red line. Data are organized by genomic grouping. Bars represent mean (±SE).

**Figure 3.**
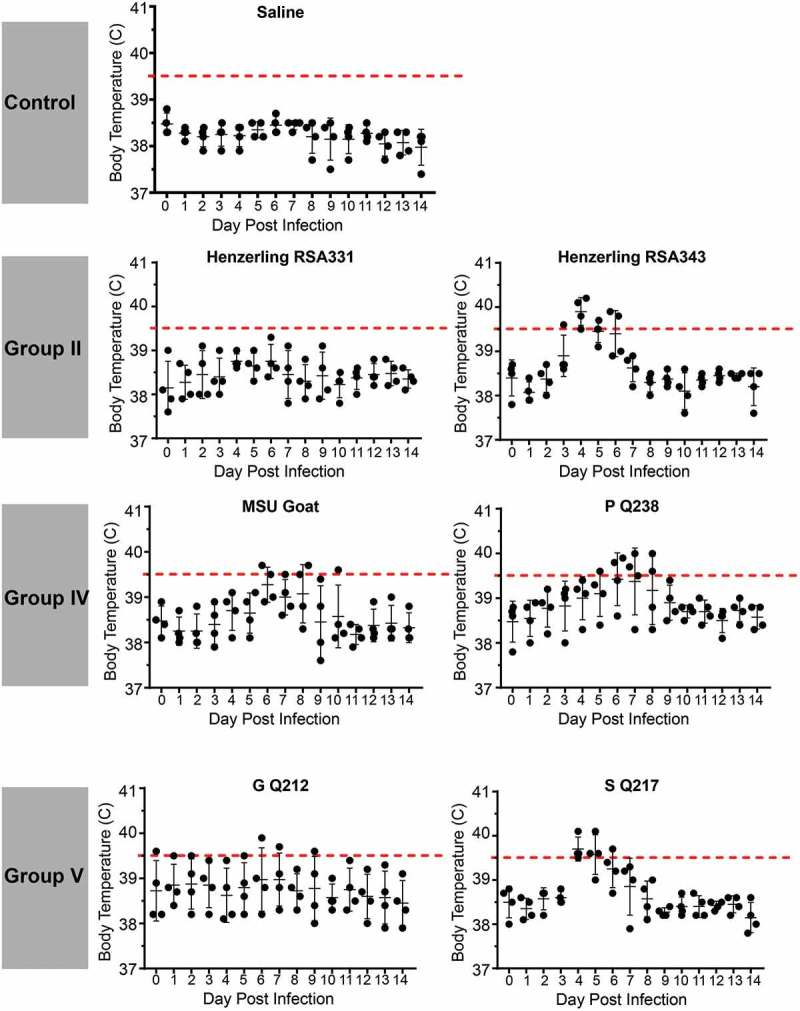
Fever responses of *C. burnetii*-infected guinea pigs in experiment 2. Body temperatures were recorded daily from the day of infection (day 0) until the day of euthanasia (day 14). Temperatures were plotted for each individual animal per time point. Fever was defined as ≥ 39.5°C and is indicated by the dotted red line. Data are organized by genomic grouping. Bars represent mean (±SE).

Guinea pigs were infected with 10^5^ GE of *C. burnetii* via intraperitoneal injection. Body temperatures were recorded daily for 14 days post infection and are presented in Figures 2 and 3. As expected, saline-treated negative controls did not develop fever (body temperature of ≥ 39.5°C) and maintained a consistent body temperature throughout the duration of each study. Similarly, the phase II LPS-expressing genomic group I NMII reference strain did not cause fever, with the exception of one animal at day 3 post infection, whose temperature was 39.7°C (Figure 2). Importantly, the temperature of this animal on the day of infection was 39.2°C, which was generally higher than that of the other animals in the study. Phase I LPS-expressing genomic group I strains, NMI and Ohio, caused fever in 3/4 animals by day 3 post infection with fever manifesting in these animals for a total of 4 and 2 days, respectively (Figure 2). Two of the animals in the NM Crazy group developed a transient, low-grade fever at day 3 post infection (Figure 2), demonstrating the attenuated virulence of this strain compared to NMI [38]. The genomic group II Henzerling strains caused divergent fever responses which correlated with their passage history and the presence of phase II LPS (Figure 3). Infection with the phase I and intermediate LPS-expressing Henzerling RSA343 caused fever in 4/4 animals by day 4 post infection while Henzerling RSA331, which produces phase I, intermediate, and phase II LPS, did not cause fever (Figure 3). The single genomic group III strain, Idaho Goat, caused fever in 3/4 animals by day 4 post infection, which began to recede the following day (Figure 2). The two genomic group IV strains caused similar responses (Figure 3): 2/4 MSU Goat-infected animals and 3/4 P Q238-infected animals developed fever that only narrowly surpassed the threshold of 39.5°C for several days (Figure 3). G Q212, a genomic group V strain, did not cause fever (mean temperature gain was not significantly increased at any time point post infection); however, a single animal with a high baseline temperature (39.6°C starting temperature) introduced a confounding factor to this group (Figure 3). The other genomic group V strain, S Q217, caused fever in 4/4 animals at day 4 post infection which subsided (in 3/4 animals) by day 6 post infection (Figure 3). The two genomic group VI Dugway strains did not cause fever, with the exception of one animal at a single time point post infection in the Dugway 7D group (39.6°C at 4 days post infection) which had a starting temperature of 38.8°C (Figure 2).

In an effort to represent temperature fluctuation among experimental groups and account for variable starting temperatures, we plotted mean change in body temperature (Supplemental Figures 1 and 2). Significant increases in body temperature compared to day 1 post infection were evidenced for NMI, Ohio, Henzerling RSA343, Idaho Goat, MSU Goat, P Q238, and S Q217-infected animals. Saline, NMII, NM Crazy, Henzerling 331, Dugway 7D, and Dugway 7E-infected animals did not significantly gain or lose body temperature throughout the duration of the study (Supplemental Figure 1). Notably, no significant changes in body temperature were observed for G Q212-infected animals (Supplemental Figure 2), further illustrating the confounding nature of high starting temperature.

### Body weight loss is minimal during infection

**Figure 4.**
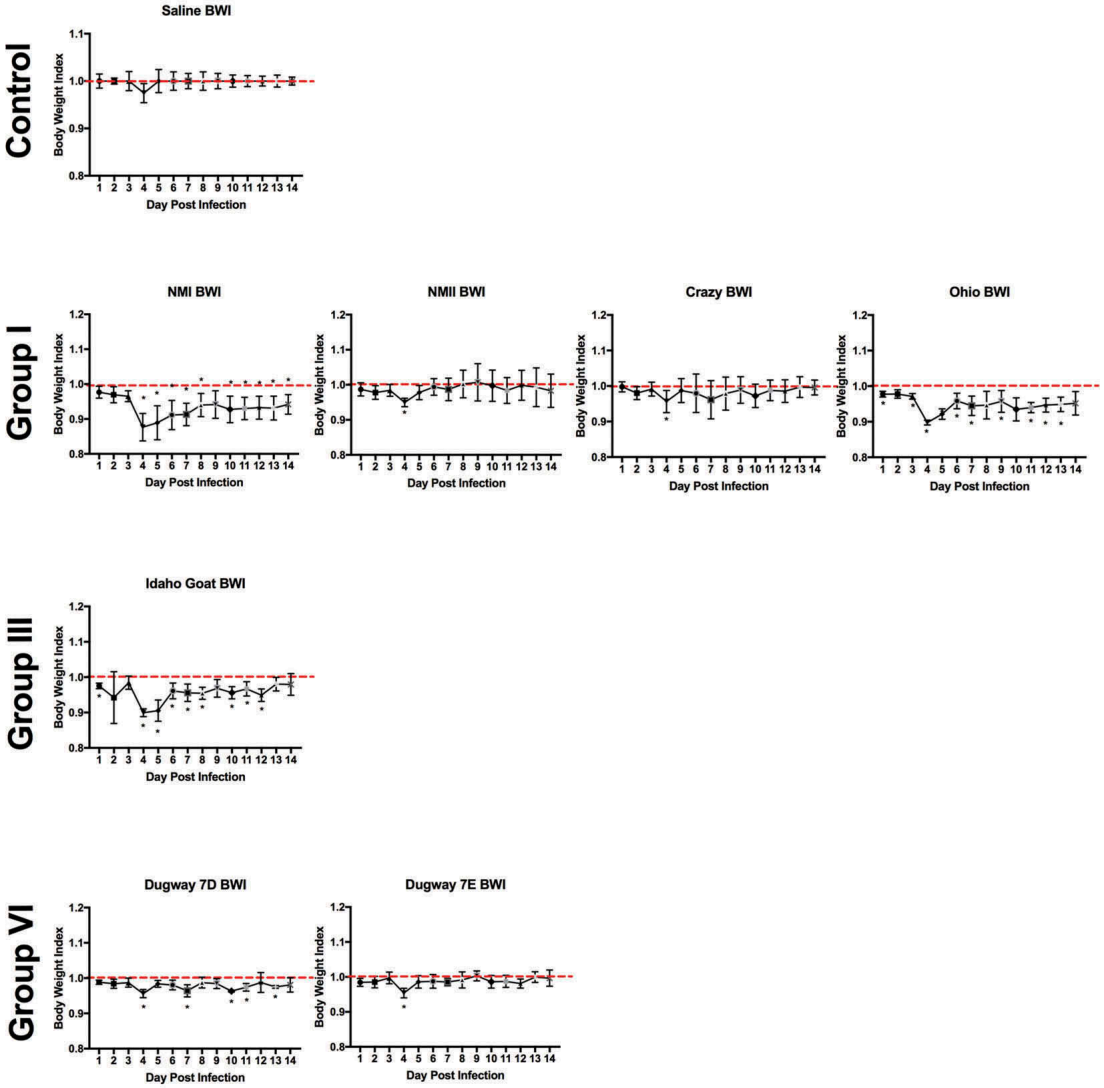
Body weight index (BWI) of *C. burnetii*-infected guinea pigs in experiment 1. Body weights were recorded daily from the day of infection (day 0) until the day of euthanasia (day 14). BWI was calculated as described in the Materials and Methods. Each symbol represents the mean value of four animals per group (±SE). Asterisk indicates p < 0.05 compared to the mean value at day 1

**Figure 5.**
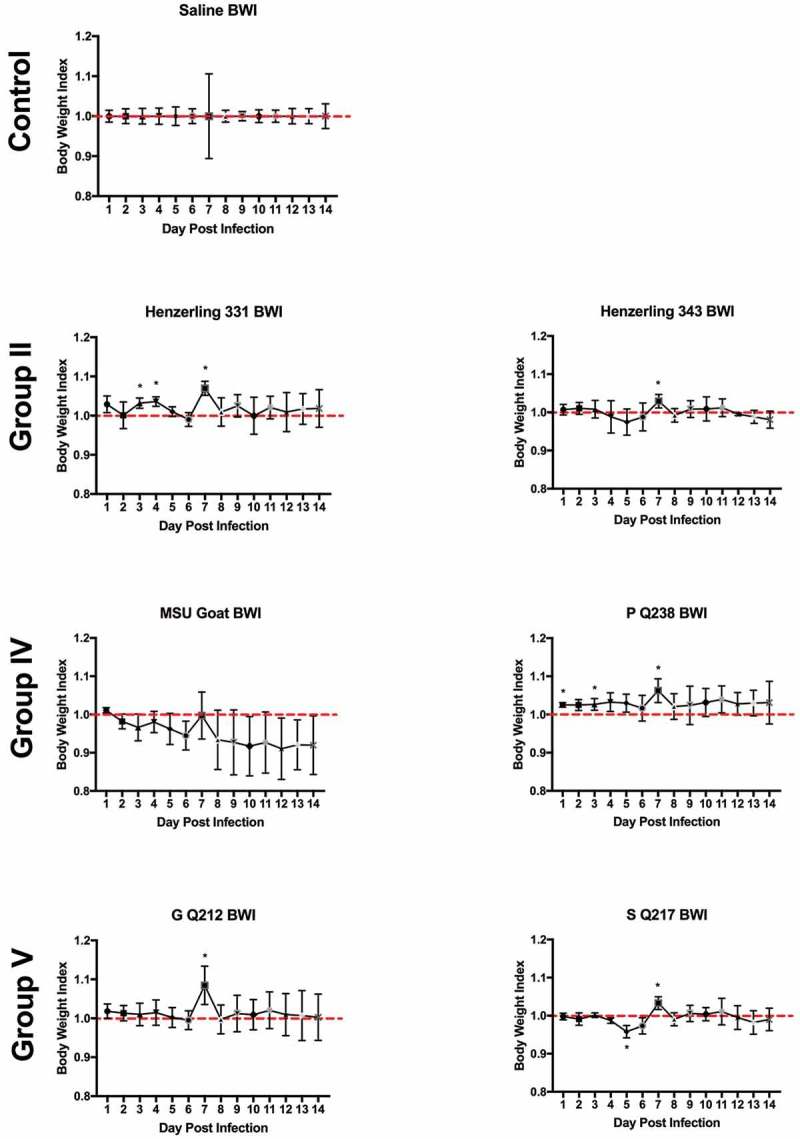
Body weight index (BWI) of *C. burnetii*-infected guinea pigs in experiment 2. Body weights were recorded daily from the day of infection (day 0) until the day of euthanasia (day 14). BWI was calculated as described in the Materials and Methods. Each symbol represents the mean value of four animals per group (±SE). Asterisk indicates p < 0.05 compared to the mean value at day 1

Body weights were monitored daily beginning on the day of infection (day 0) and ending at 14 days post infection. Body weight data are presented as percentage of body weight lost or gained compared to initial weight on the day of infection (Supplemental Figure 3(a,b)) and as body weight index (Figures 4 and 5). Generally, the duration and onset of body weight loss correlated with the severity of the febrile response exhibited by each animal. Slight body weight loss was observed following infection with NMI, Ohio, and Idaho Goat strains; weight loss was transient, beginning at day 3 post infection, and reversing by day 6 (Ohio) and day 8 (NMI) post infection (Figure 4 and Supplemental Figure 3). The onset and reversal of weight loss temporally coincided with fever with NMI, Ohio, and Idaho Goat-infected guinea pigs reaching 0.9 during early infection and exhibiting similar recovery kinetics (nearing a body weight index of 1 approximately 6 days post infection; Figure 4). One animal infected with MSU Goat lost over 10% of its original body weight which persisted throughout the duration of infection (Supplemental Figure 3; lowest body weight index: 0.85 at day 10 post infection; Figure 5). Due to the extended duration of weight loss in this animal and the absence of weight loss in the rest of the group, this weight loss may be unrelated to *C. burnetii* infection.

### Infection by C. burnetii strains from genomic groups I, II, IV, and V causes splenomegaly

**Figure 6.**
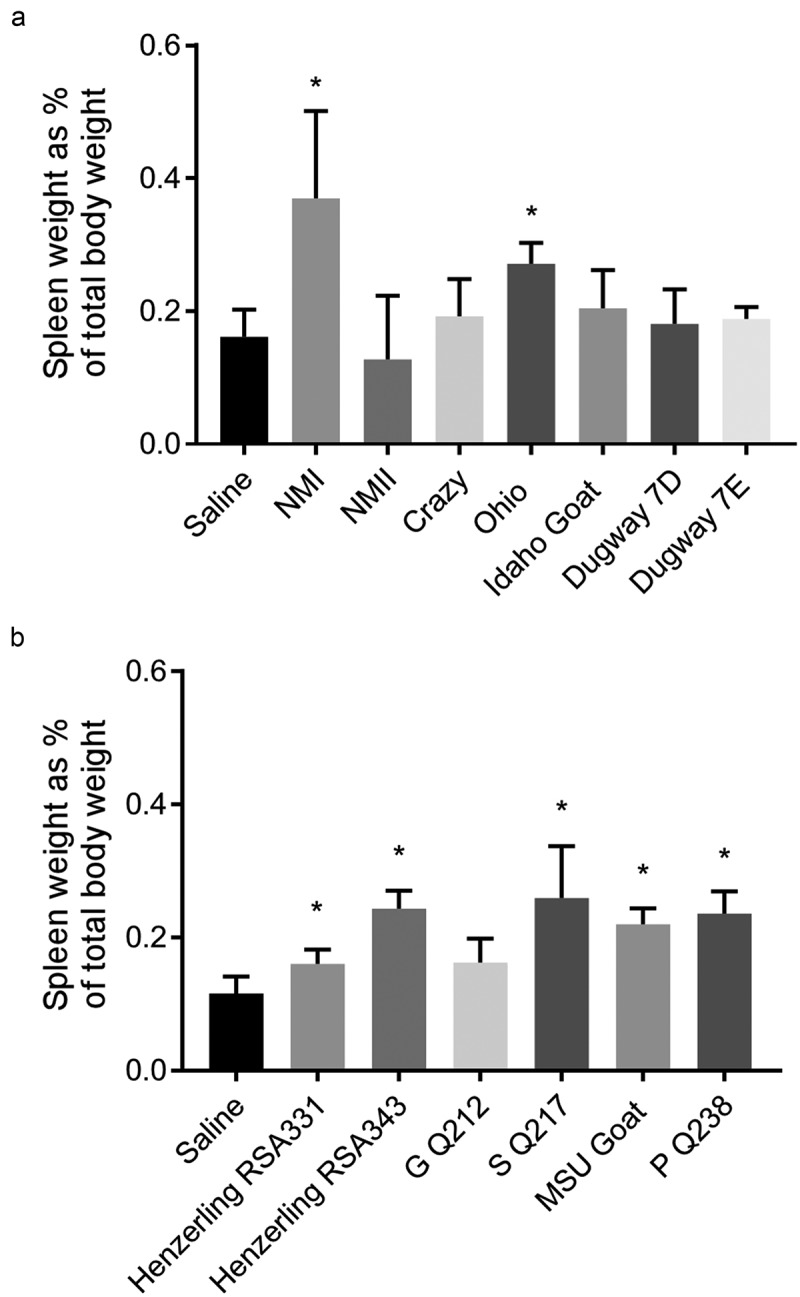
Splenomegaly in *C. burnetii*-infected guinea pigs. Spleen weights were obtained at 14 days post infection. Data are presented as a percentage of total guinea pig body weight in an effort to account for baseline variability in organ weight. Asterisk indicates p < 0.05 compared to the mean value of the saline control group.

Splenomegaly is considered a measure of bacterial pathogenicity in rodent models of *C. burnetii* infection [37]. At 14 days post infection, gross spleen weights for saline-treated guinea pigs generally ranged between 0.5–1 g (Supplemental Figure 4). When normalized to body weight, this accounted for approximately 0.1–0.2% of total body weight (Figure 6(a,b)). NMI, Ohio, Henzerling RSA343, S Q217, MSU Goat, and P Q238-infected guinea pigs exhibited significant splenomegaly (Supplemental Figure 4(a,b)). When these raw values were normalized to total body weight, these groups remained significant along with Henzerling 331 (Figure 6(a,b)).

**Figure 7.**
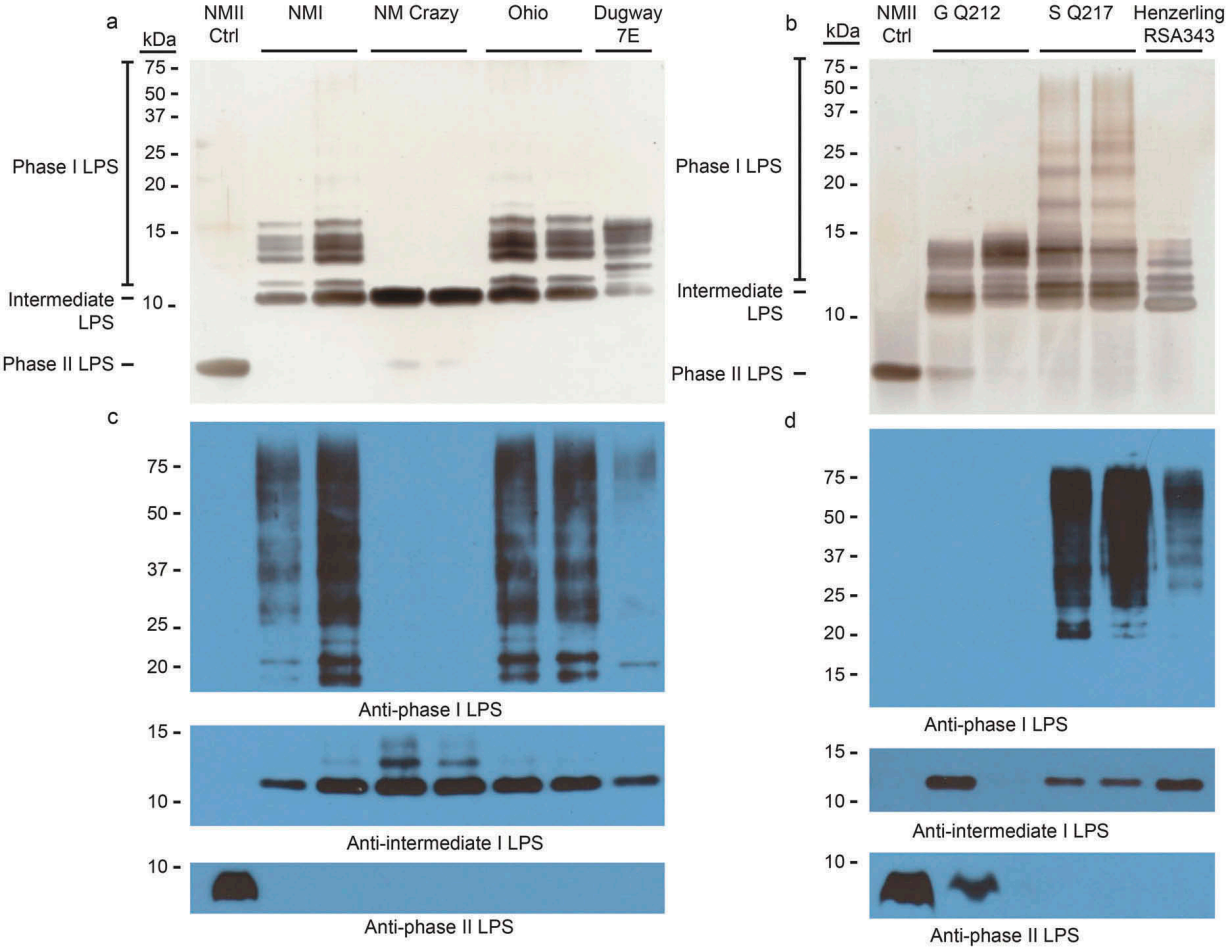
LPS profiles of *C. burnetii* strains recovered from spleen homogenates. *C. burnetii* strains were cultured by adding splenic cellular suspensions to ACCM-D. LPS was extracted from *C. burnetii* and profiled by silver stain (a and b) and immunoblot (c and d) with anti-phase I, anti-intermediate, and anti-phase II LPS antibodies for one or two representative samples per group. LPS from NMII was included as controls (ctrl).

To examine whether *C. burnetii* could be recovered from spleens, the axenic medium ACCM-D was inoculated with spleen homogenates and cultures were incubated for three one week-long passages. Bacterial growth was observed with homogenates derived from guinea pigs infected with NMI, NM Crazy, Ohio, Henzerling RSA343, Idaho Goat, G Q212, S Q217, and Dugway 7E (data not shown). NMI, NM Crazy, Ohio, Dugway 7E, G Q212, S Q217, and Henzerling RSA343 yielded sufficient growth after 2-to-3 axenic passages for visualization of LPS by silver stain (Figure 7(a,b) and immunoblot (Figure 7(c–d)). LPS profiles of recovered strains were similar to those of the initial inocula (Figure 1) with the exception of NM Crazy which displayed much less phase II LPS than the original inocula. One of the recovered G Q212 strains displayed a small amount of phase II LPS (Figure 7(b,d)) which may have been a consequence of axenic passage. Indeed, Beare et al [23]. recently showed phase I G Q212 expresses phase II LPS after only two axenic passages, unlike other strains in the study. Following similar culture conditions, *C. burnetii* growth was not observed in axenic media inoculated with lymph node homogenate or serum (data not shown). Collectively, these results show that *C. burnetii* can be recovered from infected guinea pig spleens and that passage through guinea pigs can eliminate organisms expressing phase II LPS.

### Guinea pigs infected with virulent group I strains have decreased mesenteric lymph node CD4^+^ frequency

**Figure 8.**
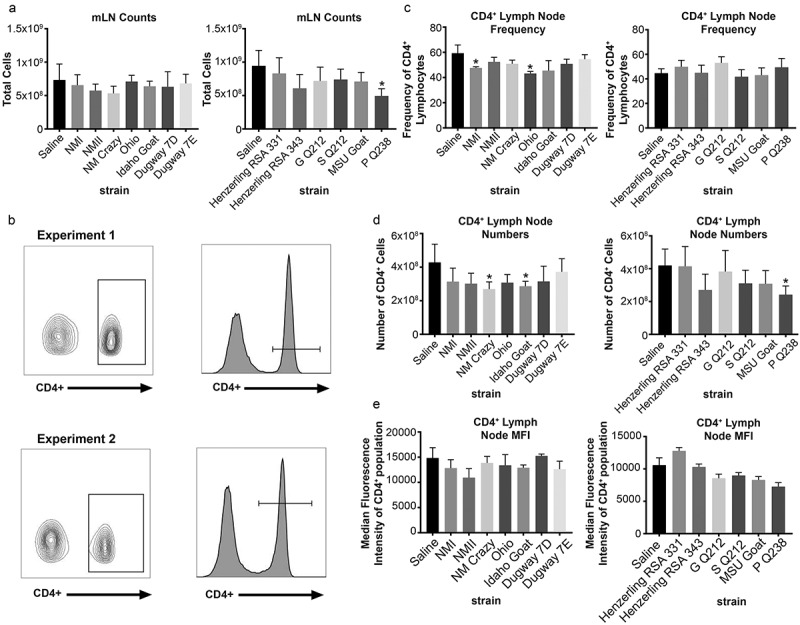
Mesenteric lymph node CD4^+^ population profile of *C. burnetii*-infected guinea pigs. Total mesenteric lymph node cellularity (a) was determined following spleen suspension. The gating strategy utilized for flow cytometric analysis of the CD4^+^ population in the mesenteric lymph node is shown in (b). CD4^+^ frequency (c), numbers (d), and median fluorescence intensity (MFI; e) are presented as the mean (±SE) of 4 guinea pigs per group. Asterisk indicates p < 0.05 compared to the mean value of the saline group.

Total mesenteric lymph nodes (mLN) cells were counted and analyzed for expression of the T cell markers CD4 and CD8 (Figure 8 and Supplemental Figure 5). Flow cytometric gating strategies are shown in Figure 8(b) and Supplemental Figure 5, respectively. mLN counts were significantly decreased in P Q238-infected animals (Figure 8(a)). CD4^+^ T cell frequency was significantly decreased in the mLNs of NMI and Ohio-infected guinea pigs (Figure 8(c)). CD4^+^ T cell numbers were significantly decreased in mLNs of NM Crazy, Idaho Goat, and P Q238-infected guinea pigs (Figure 8(d)), which was likely a consequence of decreased total cell numbers. Lastly, CD4^+^ median fluorescence intensity (MFI) was not significantly altered following *C. burnetii* infection (Figure 8(e)). The CD8^+^ T cell population was also analyzed for the same parameters (Supplemental Figure 5). CD8^+^ T cell frequency (Supplemental Figure 5b), numbers (Supplemental Figure 5c), and MFI (Supplemental Figure 5d) remained unchanged regardless of strain, with the exception of a significant decrease in CD8^+^ T cell numbers in P Q238-infected guinea pig mesenteric lymph nodes which was likely due to overall decreased mesenteric lymph node counts within this group.

### Coxiella-specific IgG antibody titers reveal divergent humoral responses and do not appear to correlate with virulence potential

**Figure 9.**
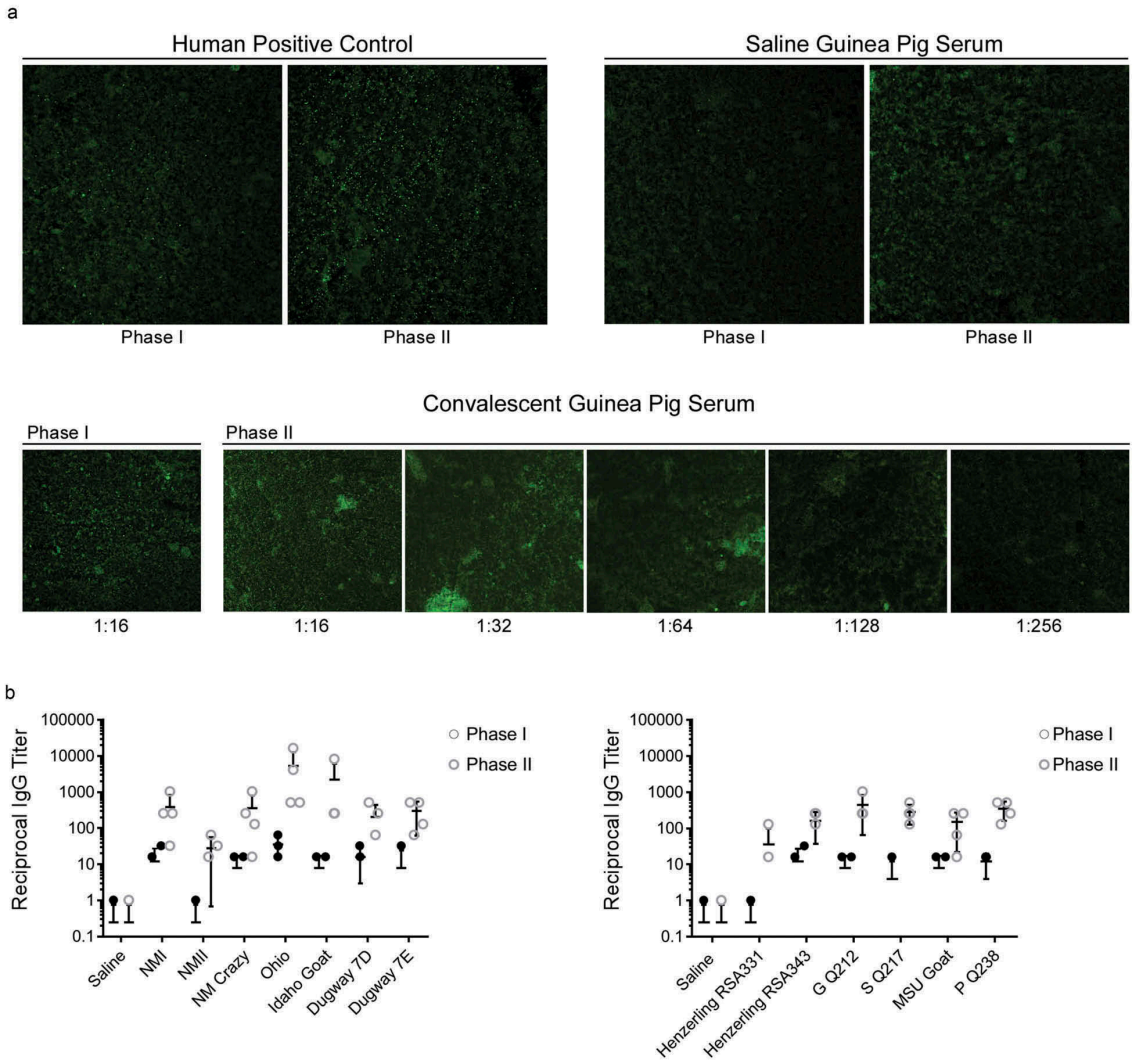
*C. burnetii* phase I and phase II IgG titers of sera from *C. burnetii*-infected guinea pigs. Serum IgG titers of *C. burnetii*-specific phase I and phase II antigens were determined via IFA. A micrograph of antigen slides treated with human positive control, guinea pig negative control (saline group), and guinea pig positive control (convalescent group, Idaho Goat infected-animal) sera are shown in (a). The reciprocal of the highest dilution that exhibited dim fluorescence (equivalent to that of the positive control at its reference endpoint titer) was defined as the endpoint titer (e.g. 1:256 for the guinea pig positive control shown in a). Strain-specific anti-*C. burnetii* phase I and II IgG titers are shown in (b). All convalescent guinea pig sera were collected at 14 days post infection.

Post infection guinea pig sera was analyzed for *C. burnetii*-specific IgG via IFA. IFA reactivity is represented by positive reactions to phase I and/or II *C. burnetii* antigens. Positive controls included human convalescent sera included in the kit and convalescent guinea pig sera from the current study (Figure 9(a)). Sera from saline-treated guinea pigs resulted in a negative reaction to both antigens, validating its use as a negative control (Figure 9(a)). IgG endpoint titers to phase I and II *C. burnetii* antigens were defined as the reciprocal of the highest serum dilution that gives obvious fluorescence as demonstrated using sera from an Idaho Goat-infected animal (Figure 9(a)). Endpoint titers are displayed in Figure 9(b). Sera from guinea pigs infected with NMII and Henzerling RSA331, which express high levels of phase II LPS, displayed IgG reactivity to phase II, but not phase I antigen. Sera from guinea pigs infected with remaining phase I strains displayed increased phase I and II IgG reactivity, with higher phase II titers, which has been noted in human acute Q fever patients [40]. Sera from Ohio-infected guinea pigs displayed the highest phase II IgG titer. Detailed IgG serologic analysis for all animals is shown in Table 2, including reciprocal mean titers, geometric mean titers, and endpoint titers for individual animals (phase I and II).

**Table 2.** *C. burnetii*-specific IgG serology.

			Reciprocal Mean IgG Titer		Geometric Mean IgG Titer		Individual Endpoint IgG Titer Phase I/Phase II			
Exp^1^	Group	Strain	Phase I	Phase II	Phase I	Phase II	Animal 1	Animal 2	Animal 3	Animal 4
1		Saline	0	0	1	1	-/-	-/-	-/-	-/-
	I	NMI	12 ± 7.7	392 ± 217.2	4.8	215.3	-/1:1024	-/1:256	1:32/1:256	1:16/1:32
		NMII	0	28 ± 13.66	1	13.4	-/1:16	-/1:32	-/1:64	-/-
		NM Crazy	8 ± 4.6	356 ± 228	4	152.2	1:16/1:1024	-/1:16	-/1:256	1:16/1:128
		Ohio	36 ± 10.1	5376 ± 3765	32	2048	1:64/1:512	1:32/1:16384	1:16/1:512	1:32/1:4096
	III	Idaho Goat	8 ± 4.6	2240 ± 1984	4	608.9	1:16/1:256	-/1:256	1:16/1:8192	-/1:256
	VI	Dugway 7D	16 ± 6.5	208 ± 115	9.5	53.8	1:32/1:64	-/-	1:16/1:256	1:16/1:512
		Dugway 7E	8 ± 8	304 ± 120.8	2.4	215.3	-/1:64	1:32/1:512	-/1:512	-/1:128
2		Saline	0	0	1	1	-/-	-/-	-/-	-/-
	II	Henzerling RSA331	0	36 ± 30.9	1	6.7	-/1:128	-/-	-/-	-/1:16
		Henzerling RSA343	12 ± 7.7	160 ± 61.3	4.8	53.8	-/1:128	1:16/1:256	1:32/1:256	-/-
	V	G Q212	8 ± 4.6	448 ± 192	4	362	-/1:256	-/1:256	1:16/1:1024	1:16/1:256
		SQ217	4 ± 4	288 ± 80.5	2	256	1:16/1:512	-/1:128	-/1:256	-/1:256
	IV	MSU Goat	8 ± 4.6	148 ± 63.1	4	90.5	1:16/1:64	-/1:16	-/1:256	1:16/1:256
		PQ238	12 ± 4	352 ± 96	8	304.4	-/1:512	1:16/1:128	1:16/1:256	1:16/1:512

^1^Exp: Experiment number

## Discussion

Here, we investigated the pathogenic potential of genetically diverse *C. burnetii* strains in a guinea pig model of infection. Genomic group I strains expressing phase I LPS (NMI and Ohio) caused the most severe disease, with the appearance of fever at day 3 in 75% of the infected animals, which is earlier than that of other virulent stains. Additionally, NMI and Ohio-infected animals reached a mean body weight index of 0.9 with comparable weight loss only observed in Idaho Goat and MSU Goat-infected guinea pigs. Splenomegaly was also observed for NMI and Ohio-infected guinea pigs and a *C. burnetii*-specific IgG response was observed following infection. The observation that genomic group I strains cause the most severe and acute disease among multiple genomic groups has been previously noted in SCID mouse and guinea pig aerosol challenge models of infection [37]. Mesenteric lymph node CD4^+^ T cell frequency was decreased in NMI and Ohio-infected animals alone, possibly correlating with virulence and potential expansion of mLN B cells, although this remains to be investigated. The group II Henzerling RSA343 strain, also associated with acute human disease, caused fever in 100% of the animals, yet appeared to be less virulent than group I strains (based on absence of weight loss and shorter fever duration). The only genomic group III strain tested, Idaho Goat, appears to be nearly as virulent as the genomic group I strains, only falling short due to the absence of splenomegaly. This strain has not been associated with human disease, yet it retains the QpH1 plasmid like the group I and II strains. Genomic group IV strains are associated with persistent focalized infections (P Q238 is from a human Q fever endocarditis patient [13]) and these strains appeared to be mildly virulent in our model, with animals exhibiting low grade, late-onset fevers, splenomegaly, and elevated *C. burnetii*-specific IgG titers in the absence of weight loss. Genomic group V strains are also associated with persistent, focalized infections [13]. Surprisingly, G Q212 and SQ217 caused different degrees of clinical disease in our model, with G Q212 failing to induce significant fever in 75% of animals, and animals displaying a lack of mean body weight loss and splenomegaly in the presence of elevated *C. burnetii*-specific IgG titers. S Q217 appears to be more virulent than G Q212, causing fever, splenomegaly, and elevated *C. burnetii*-specific IgG titers in the absence of weight loss. G Q212 was isolated from a human heart valve (Q fever endocarditis patient) and S Q217 originated from a human liver biopsy (Q fever hepatitis patient) [13]. The origin of these strains, paired with the intraperitoneal route of infection utilized in our model, may have influenced the severity of illness caused by each strain. In a separate study, S Q217 caused body weight loss of a slightly greater magnitude in CB-17 mice and more elevated and sustained fever in guinea pigs than G Q212 [37]. Lastly, the genomic group VI strains, Dugway 7D 77-80 and 7E 65-68, appeared avirulent due to the absence of any clinical presentation of disease. Interestingly, these strains produce phase I LPS needed for virulence. Thus, they likely lack additional factors required for virulence or produce factors that attenuate virulence. Dugway strain avirulence has been previously reported in murine and guinea pig models of infection [11,37]. Of note, the magnitude of *C. burnetii*-specific IgG levels did not appear to correlate with the severity of disease caused by each strain. In acute Q fever patients, phase II IgG levels rise more quickly than those of phase I IgG [40–43]. In accordance with this observation, mean phase II IgG titers were higher than phase I titers for each group at 14 days post infection in our model, illustrating another physiological correlate with human disease.

Our findings support the hypothesis that strains within defined genomic groups exhibit similar virulence that may be distinct from virulence of other genomic groups [7,13]. The number of strains examined from each genomic group precludes statistical analysis of virulence traits between groups. Nonetheless, our extensive 13-strain study clearly shows trends in pathogenesis that are likely manifested by genotype and LPS content [18,37]. This correlation is particularly evident with respect to virulent group I strains associated with acute Q fever in human patients. Indeed, group IV and V strains P Q238, G Q212, S Q217, and MSU Goat, isolated from humans and animals with persistent focalized infections, were less virulent than group I strains (NMI and Ohio), displaying shorter fevers of less magnitude and no body weight loss. Strikingly, the group II Henzerling RSA343 strain did not cause disease as severe as NMI and Ohio, in spite of its origin (human acute Q fever patient) and shared plasmid (QpH1). This observation suggests that factors beyond plasmid type influence virulence in a guinea pig intraperitoneal infection model. As expected, all strains exhibited low phase I titers as the serologic response to O-antigen is delayed relative to the response to phase II protein antigens. Typically high phase I titers are not evidenced until at least 20 days post infection [17,19]. Notably, avirulent strains containing bacteria that produce significant amounts of phase II LPS (e.g. NMII and Henzerling RSA331) induced lower phase II antigen-specific IgG titers than avirulent strains producing solely phase I LPS (e.g. G Q212, Dugway 7D, and Dugway 7E). One possibility for this result is that avirulent NMII and Henzerling RSA331 are more defective for growth *in vivo* due to the proinflammatory nature of organisms producing phase II versus phase I LPS, potentially making these strains more susceptible to inflammatory effects and clearance [44].

*C. burnetii* LPS phase transition is fundamentally important in virulence [18]. Strains producing both phase I and II LPS can be highly attenuated, if not avirulent due to truncated LPS expression [18,21,45]. *In vitro* passage history is often poorly catalogued; thus, one frequently cannot assume LPS phase type. Hence, we profiled the LPS of all inocula prior to animal infection. This process represents a crucial step in *C. burnetii* virulence studies where LPS phase influences experimental outcome [18,21,45]. By including strains expressing different forms of LPS, we were able to analyze the effects of LPS during infection along with the fate of LPS following infection. Infection with the isogenic strains NMI, NM Crazy, and NMII was particularly informative, as the only difference between these strains is LPS length. Here and elsewhere [18], infection with these strains demonstrated a direct correlation between LPS length and virulence. NMII, a clonal phase II strain that only expresses phase II LPS, was not recovered from the spleens of infected animals. Notably, this strain did not cause fever, weight loss, or splenomegaly, yet phase II IgG levels were elevated following infection. These data suggest that this strain is cleared by the host during an asymptomatic infection. Our laboratory passaged NM Crazy RSA514 strain expresses both intermediate and phase II LPS and was recovered from the spleens of two animals. The recovered bacteria expressed dramatically less phase II LPS than the initial inoculum. This phenomena was serologically observed in the initial description of phase variation [19]. This process of phase II reduction within the bacterial population *in vivo* is likely related to increased clearance of phase II variants in the mixed inocula [19].

The inclusion of high-pass Henzerling RSA331 (substantial phase II LPS) and low-pass Henzerling RSA343 (undetectable phase II LPS) strains allowed for the examination of the role of LPS phase *in vivo* produced by isogenic group II strains. Henzerling RSA331 (high-pass) did not cause fever while Henzerling RSA343 (low-pass) did. Both strains caused significant splenomegaly; however, only Henzerling RSA343 was recovered from the spleen of an animal. Again, the failure of a strain expressing both phase I and phase II LPS (Henzerling RSA331) to cause clinical disease and to be recovered from the spleen of an infected animal may reflect the proinflammatory nature of phase II organisms that also limits growth of phase I organisms within an inoculum *in vivo* [44]. Importantly, not all phase I LPS-expressing bacteria in this study caused disease (such as the Dugway strains). This observation supports the concept that pathotype-specific virulence can be mediated by virulence or anti-virulence factors [18]. Additionally, splenic bacterial recovery did not directly correlate with virulence. This may have resulted from varying axenic growth efficiencies, variability in spleen homogenization, or altered bacterial clearance *in vivo*.

The guinea pig Q fever model is physiologically relevant chiefly due to an infectious dose-febrile response similar to that of humans and non-human primates [18,37,46–48]. This febrile response is not present in the commonly utilized mouse model of infection, with the primary indicator of disease being splenomegaly [37]. Beyond similarities in the fever response between humans exposed to *C. burnetii* via aerosol and guinea pigs exposed via aerosol and intraperitoneal injection, additional similarities exist with respect to infectious dose ranges, incubation periods, and serologic responses [49]. The virulence of several *C. burnetii* strains has been evaluated in a guinea pig model of aerosol infection and the febrile patterns mirror those reported here [37]. Generally, the appearance of fever is earlier following intraperitoneal infection; yet, the duration and magnitude of fever are remarkably similar in both models. Collectively, these observations indicate that the intraperitoneal route of infection is a valid model for virulence assessment that is accessible and sensitive.

Altogether, our data support the hypothesis that genomic groups represent pathotypes of *C. burnetii* with specific virulence potentials. These findings are consistent with data previously reported by Russell-Lodrigue et al. [37] in rodent models of infection. Genomic sequencing of *C. burnetii* has identified unique genes between genomic groups, some with potential effector functions that may influence virulence [13,50,51]. Of note, the Dugway strains (genomic group VI) exhibit the largest genome of all *C. burnetii* strains but are avirulent; hence, genome reduction may be associated with increased *C. burnetii* virulence. These studies highlight the role of *C. burnetii* genomic content in virulence potential. The guinea pig intraperitoneal model of infection constitutes a robust system to study strain-specific genes and their roles in *C. burnetii* pathogenesis.
